# Diseases of the Skin

**Published:** 1893-07

**Authors:** 


DISEASES OF THE SKIN, by Chas. C. Ransom, M. D., Philadelphia, Lea Bros. & Co. Price, $1.00.

One of the student's quiz series, nicely gotten up and well illustrated, and giving at the end of the book the classification adopted by the American Dermatological Association, together with a number of selected formulae, for internal and external use.

